# Human tooth pulp anatomy visualization by 3D magnetic resonance microscopy

**DOI:** 10.2478/v10019-012-0018-y

**Published:** 2012-03-06

**Authors:** Dusan Sustercic, Igor Sersa

**Affiliations:** 1 Department of Prosthodontics, Medical Faculty, University of Ljubljana, Ljubljana, Slovenia; 2 Jožef Stefan Institute, Ljubljana, Slovenia; 3 EN-FIST Centre of Excellence, Ljubljana, Slovenia

**Keywords:** MR microscopy, dental pulp anatomy, endodontic treatment, 3D visualization

## Abstract

**Background:**

Precise assessment of dental pulp anatomy is of an extreme importance for a successful endodontic treatment. As standard radiographs of teeth provide very limited information on dental pulp anatomy, more capable methods are highly appreciated. One of these is 3D magnetic resonance (MR) microscopy of which diagnostic capabilities in terms of a better dental pulp anatomy assessment were evaluated in the study.

**Materials and methods:**

Twenty extracted human teeth were scanned on a 2.35 T MRI system for MR microscopy using the 3D spin-echo method that enabled image acquisition with isotropic resolution of 100 μm. The 3D images were then post processed by ImageJ program (NIH) to obtain advanced volume rendered views of dental pulps.

**Results:**

MR microscopy at 2.35 T provided accurate data on dental pulp anatomy *in vitro*. The data were presented as a sequence of thin 2D slices through the pulp in various orientations or as volume rendered 3D images reconstructed form arbitrary view-points. Sequential 2D images enabled only an approximate assessment of the pulp, while volume rendered 3D images were more precise in visualization of pulp anatomy and clearly showed pulp diverticles, number of pulp canals and root canal anastomosis.

**Conclusions:**

This *in vitro* study demonstrated that MR microscopy could provide very accurate 3D visualization of dental pulp anatomy. A possible future application of the method *in vivo* may be of a great importance for the endodontic treatment.

## Introduction

Magnetic resonance imaging (MRI) is nowadays a well-established imaging modality that is used in various medical fields as well as in material science.^1^,^2^ Among medical MRI applications it is also an emerging field of MRI applications in dentistry which include: diagnosis of temporomandibular joint pathological changes, inflammatory conditions of the facial skeleton, examination of salivary glands, maxillary sinuses, masseter muscles, detection of early bone changes such as neoplasm, fractures, inflammatory conditions, as well as imaging of mouth floor and tongue.^3^–^12^ Furthermore, some attempts were made to image hard dental tissues, such as enamel, dentin and cementum.^13^–^15^

MRI in dental applications can be divided into imaging of soft dental tissues (dental pulp and periodontal tissues) and of hard dental tissues (enamel and dentin). Hard dental tissue imaging is in particular challenging as the amount of water in the microstructure of enamel and dentine is low and T_2_ relaxation times of water in the tubules are very short, of the order of a millisecond.^16^, ^17^ Both effects significantly reduce the MRI signal and make imaging of these tissues practically impossible with the use of standard imaging techniques, such as spin-echo (SE) or gradient-echo (GE). However, hard dental tissues can still be imaged by MRI using special techniques that were designed to acquire MR signal from samples with short T_2_ relaxation times. Such techniques are single point imaging (SPI), SPRITE and stray field MR imaging (STRAFI).^18^–^20^ In caries lesions properties of hard dental tissues are significantly altered. A demineralization process in the lesions results in an increase of porosity and with it associated increase of water concentration and prolongation of T_2_ relaxation time of water in dentin.^21^ This makes detection of caries lesions possible using standard MRI techniques, such as T_1_-weighted MRI.^4^,^22^ One of most recent MRI studies demonstrated that caries affected also dental pulp by changing its diffusion properties.^23^

As opposed to hard dental tissues, soft dental tissues have higher water content and much longer T_2_ relaxation times. Therefore, MRI imaging of these tissues by standard MRI techniques, such as 2D or 3D spin-echo or gradient-echo, is possible. While today’s MRI technology can provide clinically useful images of soft tissues *in vitro*^24^, *in vivo* application of the method is still a challenge.^25^ Relatively a high MRI signal of soft dental tissues enables also high-resolution imaging of a dental pulp anatomy. An early attempt in this direction was done by Lockhart *et al*..^15^ In the study a strong 9.4 T magnetic field was used to obtain MR images of the pulp chamber *in vitro* and to visualize the tooth outline. Differences of signals from different anatomical regions of the tooth were detected. Tanasiewicz demonstrated the use of the 3D spin-echo MR imaging technique as a tool to visualize the inner space in the root canal system during the prosthodontic procedure for post preparation.^26^

In everyday clinical practice, for a successful endodontic treatment that includes the removal of all infected material in the dental pulp, it is extremely important to have an accurate assessment of the pulp chamber anatomy and of root canals. Large diversities of pulp chamber and pulp canal shapes are present within the same tooth group. In addition, teeth of the same group may have different number of pulp canals. Canal irregularities such as anastomosis, small lateral canals and canal splitting are also frequently observed.^27^ These irregularities make the endodontic therapy difficult and its outcome less predictable unless a precise dental pulp anatomy is known before the treatment. In a standard clinical procedure, the pulp anatomy is assessed from tooth radiographs, which are only 2D projections of a tooth and can show only hard dental tissues and not the soft pulp tissue. Therefore, anatomy of the dental pulp and root canals on X-ray films is presented only indirectly as signal voids (empty spaces) inside the tooth. These problems could be overcome by a technique that would enable visualization of soft dental tissues.

The aim of our work was to demonstrate that magnetic resonance (MR) microscopy is a powerful tool for visualization of soft dental tissues and can be used for a precise assessment of the dental pulp anatomy. Our study was performed on extracted human teeth that were imaged in 3D by high-spatial-resolution magnetic resonance imaging (MR microscopy). Although the radiographic examination is still a diagnostic method of choice in dentistry, MR imaging may take its place in future due to its several advantages. MRI can visualise soft dental tissues and is harmless as it does not involve any ionizing radiation. This is especially important when repetitive examinations are required.

## Materials and methods

Twenty extracted human teeth were used in this study: seven premolars and thirteen molars. The teeth were extracted due to orthodontic therapy (four premolars), due to surgical intervention (two premolars and nine molars) and due to periodontal problems (one premolar and four molars). The visual inspection of the extracted teeth showed that one premolar had composite filling on occlusal and distal plate, one had local demineralization process, two molars had caries lesions on the occlusal and a proximal face, and one molar had amalgam filling on the occlusal plate. Remaining teeth were intact. The teeth were immersed in physiological solution immediately after the extraction and stored in a low temperature environment (4°C). MRI imaging of each tooth in the study was started within 12 h after the tooth extraction to avoid autolytic changes in the pulp that may affect its MR image. To prevent the tooth desiccation during the experiment all the teeth were protected by either a thin layer of paraffin or tube sealing wax. Tooth coating with sealing wax was very convenient as it enabled visualization of the tooth outline. Namely, the imaging method used in the study enabled the detection of sealing wax and not of paraffin as it had too short MR signal.

**FIGURE 1:**
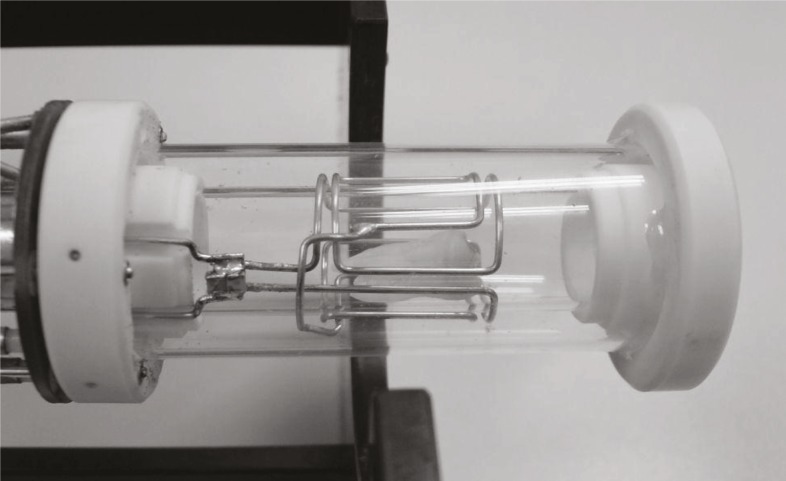
A 15 mm RF coil insert for a MR microscopy probe. A human molar is placed inside the coil.

MRI of teeth was performed on a system for MR microscopy consisting of a TecMag MR spectrometer and a 2.35 T horizontal bore Oxford superconducting magnet equipped with a Bruker MR microscopy probe with maximum imaging gradients of 300 mT/m. The probe had RF inserts of various sizes. All experiments were performed using a 15 mm RF insert (Figure 1). To obtain high spatial resolution and a good signal to noise ratio of soft dental tissues the teeth were scanned by 3D spin-echo imaging technique. In all experiments imaging field of view was equal to 25 mm in the tooth axial direction and was equal to 12.5 mm in both perpendicular directions, imaging matrix was 256 by 128 by 128, which yielded imaging resolution of 100 μm in all three spatial directions. Other imaging parameters were echo time 2.4 ms and repetition time 600 ms. Images were acquired with eight signal averages to improve their signal to noise ratio. The total scan time was 22 hours.

Acquired raw image data sets were reconstructed by NTNMR software (TecMag, Houston TX, USA) to obtain 3D images of teeth. The 3D images were then post processed by the ImageJ program (NIH, Bethesda MD, USA) using VolumeJ plugin (University of Iowa Hospitals and Clinics, Iowa, USA) to calculate advanced volume rendered views to dental pulps.

The investigators followed recommendations of the Helsinki Declaration (1964, with later amendments) and of the European Council Convention on Protection of Human Rights in Bio-Medicine (Oviedo 1997).

## Results

**FIGURE 2:**
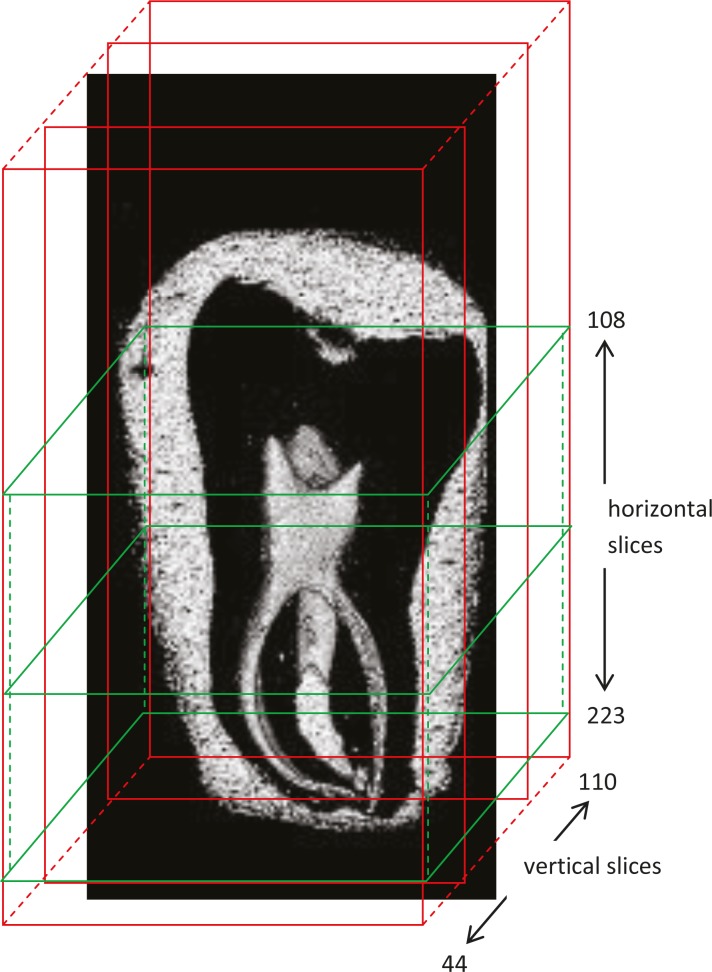
Volume rendered image of an extracted human tooth. Image orientation of the tooth is from mesial to distal part. Mesio-lingual and mesio-distal canals are in front of the image, at the back it is the distal canal. Hard dental tissues (dentine and enamel) produce no detectable MR signal due to a low water content and short T2 relaxation time. However, the outline of hard dental tissues can still be seen as a signal void region between the surface wax coating and the pulp inside the tooth. Red and green lines indicate positions of vertical and horizontal slices across the tooth.

**FIGURE 3:**
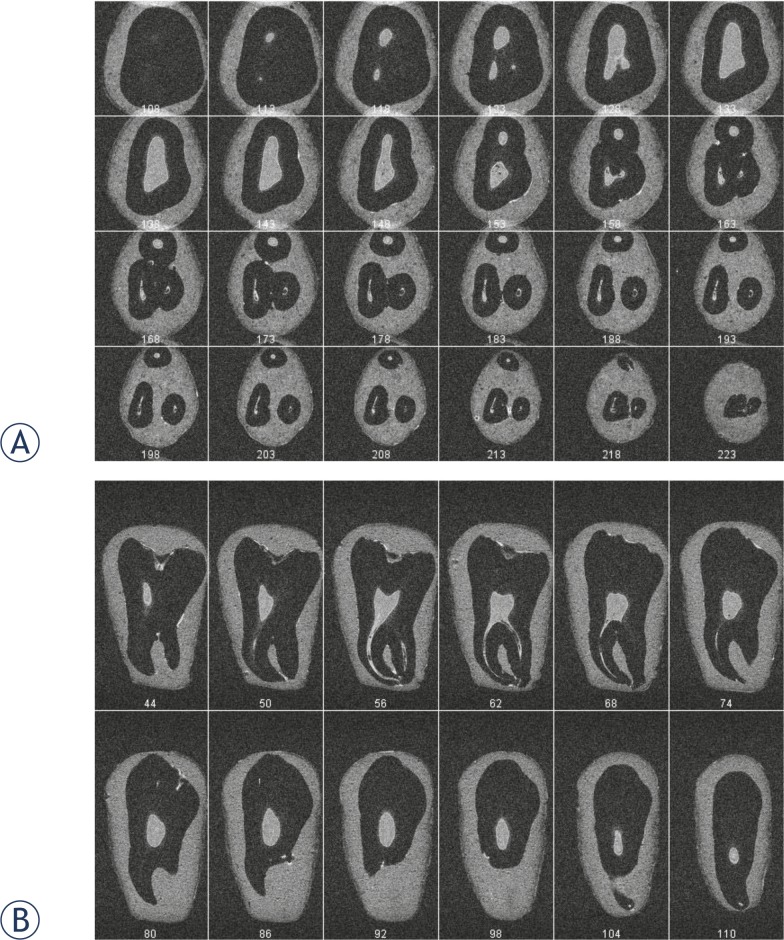
Images of consecutive 24 horizontal (A) and 24 vertical (B) slices across the dental pulp in Figure 2. The images are subsets of the 3D T1-weighed MR image of the pulp acquired using imaging matrix 256 × 128 × 128 and isotropic image resolution of 100 μm; numbers at the bottom of each image are slice indices. The pulp anatomy in horizontal plane is visualised from the coronal parts to the apical foramina. In horizontal slices, shape and volume of the pulp chamber as well as the number and shape of root canals are presented. The cross section of the mesio-buccal and distal canal is spherical in contrary to the mesio-lingual canal, which is more oval and kidney-shaped. A precise inspection of the mesio-lingual canal in slice 65 shows that its cross section is elongated from mesial to distal direction and that it could consist of two canals. Vertical slices are convenient for tracking of the course of single root canals.

A 3D MR microscopic image of an extracted human tooth (lower second molar) is presented in Figure 2. The tooth orientation in the image is from mesial to the distal part. Mesio-lingual and mesio-distal canals are in front of the image, while at back it is the distal canal. The outline of the tooth is visualised by covering its surface with sealing wax with a detectable MR signal. A dark region between the inner dental pulp and the outer layer of the sealing wax coating corresponds to hard dental tissues (enamel and dentine). In the image, hard dental tissues appear dark as they contain low amount of free water, which has in addition also very short T_2_ relaxation time. Therefore, hard dental tissues yielded an NMR signal undetectable by the standard spin-echo method. In contrary to hard dental tissues, the dental pulp can be clearly seen. The pulp is a soft dental tissue and it yields a MR signal detectable by the standard spin-echo method due to a high amount of free water and its relatively long T_2_ relaxation time. Vertical and horizontal schematic lines in Figure 2 represent positions of vertical and horizontal slices across the tooth that are shown in Figure 3.

Figure 3 depicts anatomy of the tooth from Figure 2 in consecutive horizontal (A) or vertical (B) slices. In Figure 3A, pulp anatomy in horizontal plane is visualised from coronal parts (slice 108) to the apical foramina (slice 223). A progression of the pulp chamber shape and volume is presented from slice 113, where distal and a mesio-lingual diverticle are first noticed, to slice 138, where the maximal volume is reached. The mesio-buccal diverticle is visible in slice 123. The pulp volume begins to decline from slice 138 to slice 158 where the pulp chamber ends and root canals start. Therefore, the entire pulp chamber is presented in slices from 108 to 158. The number and shape of root canals are best seen in slice from 158 to 223. From the canal start in slice 158 to the apical end in slice 223, it is possible to track the path of each individual canal. In slice 158 three canals can be seen: a mesio-lingual, mesio-distal and distal canal. The cross section of the mesio-buccal and distal canal is spherical in contrary to the mesio-lingual canal, which is more oval and kidney-shaped. A more careful inspection of the mesio-lingual canal in slice 168 shows that its cross section is elongated from a mesial to distal direction and that it could consist of two canals. The second mesio-lingual canal is smaller and it could be interconnected with the first mesio-lingual canal. Pulp anatomy in consecutive vertical slices is shown in Figure 3B in slices from 44 to 110. The most prominent part of the pulp chamber is shown in slice 44 (in horizontal slice 133 and 138 in Figure 3A). The course of the mesio-buccal root canal can be seen in consecutive vertical slices for 56 to 68.

**FIGURE 4:**
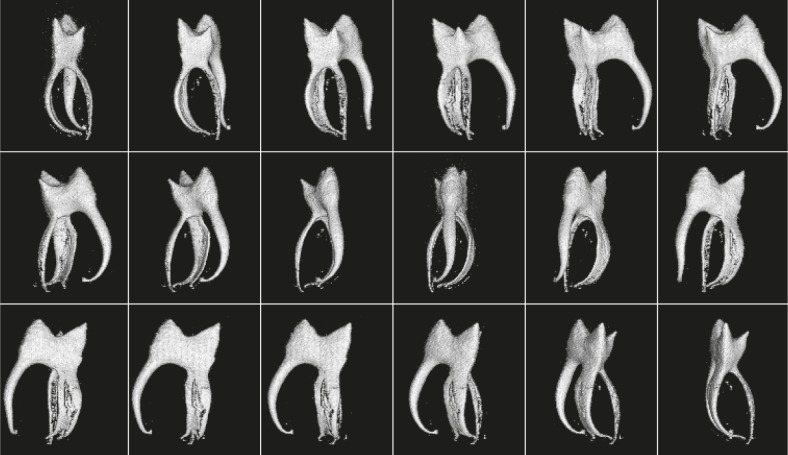
Volume rendered images of the dental pulp from Figure 3 in 18 different viewpoints 20° apart around the vertical axis. The shape of the pulp chamber, extent of pulp diverticles and the number of pulp canals are visualized with a high accuracy. Volume rendered images clearly demonstrate the complex anatomy of the dental pulp dental pulp and its unique shape. The presence of fourth root canal is obvious. The mesio-lingual canal is consisted of two interconnected canals. The wrapping of all canals toward the central vertical axis can also be seen.

The same 3D MR microscopy data, as already shown in Figure 3, can be used to calculate volume rendered views of the dental pulp from various viewpoints. An example of this is shown in Figure 4 where view points to the tooth from Figures 2 and 3 are 20° apart around the vertical axis. Shape of the pulp chamber, spatial configuration of a root canal system, extent of pulp diverticles and the number of pulp canals are visualized in the volume rendered images with an even higher accuracy than in consecutive 2D slices (Figure 3). The presence of the fourth root canal, which was in a single projection technique only a speculation, is now obvious. A careful inspection of rotated 3D images of the dental pulp clearly demonstrates dental pulp complex anatomy and its unique shape. The mesio-lingual canal is consisted of two interconnected canals. The wrapping of all canals toward the central vertical axis is also apparent. The curvatures are pronounced at the apical third of all canals. The distal canal is twisted not only to the centre of the apical part but at the same time also to the buccal direction of the tooth (the first image in Figure 4). Volume rendered images enabled visualization of the root canal system along with tracking of each individual root canal from the pulp chamber to the apical end. The accurate determination of the number of root canals was possible as well.

The used method performed equally well also in other teeth included in the study that had various anatomical features. In all cases the visualized tooth anatomy was precise and allowed the anatomy assessment of diagnostic relevance.

## Discussion

Results of our *in vitro* study at 2.35 T demonstrated that high-resolution 3D MR imaging of a dental pulp with high accuracy is feasible and that this technique has numerous advantages over standard radiographic imaging of teeth. Firstly, the radiographic technique requires the use of harmful X-ray radiation, while there is no harmful radiation in MRI. The only limiting factor with respect to safety issues is the specific absorption rate (SAR), which leads to tissue heating. Secondly, as opposed to radiographic imaging, which is a 2D projection technique, MRI is not a projection technique and can acquire images of the sample in a sequence of 2D slices or as a 3D data set with an isotropic spatial resolution. The path of X-rays in dental radiographs is always in bucco-oral direction (X-ray path is perpendicular to bucal plane of the tooth) so that overlapping of tooth details almost always occurs. In addition, 3D image datasets contain much more information compared to radiographic 2D projections. 3D images can be converted into a projection if all slices of identical orientation are summed together. However, 3D image data sets enable many other advanced image processing operations, such as reconstruction of image slices across the sample in an arbitrary orientation or volume rendering operations. Thirdly, MRI can detect signal of soft tissues, so it is very convenient for imaging of the dental pulp. The image of the pulp cannot be directly acquired by radiographic imaging. This can directly detect only hard dental tissues, whereas the pulp anatomy can be estimated only indirectly form signal voids between hard dental tissues. In that sense MRI produces images of a reversed contrast as compared to the radiographic technique (dark solid tissues and bright soft tissues). The fact that a dental pulp produces a strong MRI signal can be used for the assessment of the dental pulp anatomy, either in a sequence of 2D slices or by volume rendered projections of the pulp. As it is shown in our study (Figure 4), the latter has several advantages in terms of more precise observation and better understanding in comparison with the standard X-ray radiography.

MRI and X-ray radiography have an inherently different signal origin. In MRI, the signal originates in protons of liquids in a sample, while in X-ray the signal is a consequence of X-ray absorption on their travel across the sample. The X-ray absorption depends on a tissue density. It is higher in hard dental tissues, which have a high mineral content and it is lower in tissues with a low mineral content. As soft dental tissues have a very low mineral content, X-rays travel through them practically without any absorption so that soft dental tissues represent signal voids or an empty space inside the tooth in radiographs. As X-rays can only detect differences in film exposure, the detection of a decay or caries lesion in enamel or dentin is very difficult if it is surrounded by a massive well mineralised tissue.

The projection nature of radiography, which is its major limitation, can be overcome by CT or micro-CT scanning, which can produce images of individual slices across the sample. In that sense CT is a true imaging modality and is not just a projection technique. However, image slices are limited in orientation by the rotation axis of the X-ray source and detectors. In addition, contrasts characteristics of CT are similar to that of radiographic imaging (bright solid tissues and dark soft tissues). MRI is not limited in orientation of imaging slices. These can be acquired in any spatial orientation.

Large diversities in dental pulp anatomy are known from literature.^28^ Using MR microscopy, size and shape of the tooth pulp chamber, the number, size and shape of root canals can be clearly visualised. Conventional tooth radiographs show only 2D projection of a tooth in the bucco-lingual direction in which the third and fourth canal cannot be seen at all. The superposition of pulp structures, which lay in the same bucco-lingual plane are the cause for the lack of accuracy of classical X-ray radiographs. In our example the fourth canal is smaller and originates at almost the same part of the pulp chamber as mesio-lingual canal, it is very difficult to detect it. Because of this, in everyday clinical practice, teeth with anatomical particularities are likely to be wrong diagnosed, which could jeopardise their endodontic treatment. The difficulty of finding the fourth canal is well demonstrated also in Figure 3A, where horizontal slices across the second molar are shown. The fourth canal is considerably smaller and is interconnected with the mesio-lingual canal from the pulp chamber, where it originates to the apical part of the tooth. This anatomical feature is clearly presented by the sequence of volume rendered 3D images of the dental pulp (Figure 4).

In our *in vitro* experiments teeth were coated with a material (thin layer of paraffin or tube sealing wax) that prevented desiccation of the tooth during imaging. This was important as desiccation could result in production of void spaces at the dentin-pulp interface. In addition, small gas bubbles could also be produced at the interface as a result of autolytic changes of the pulp tissue. These effects can be seen in Figure 4 as areas of roughened surface in volume rendered images of root canals.

Presently, a conventional radiographic examination is still much cheaper and more available than a clinical MRI examination. Conventional radiography provides images of teeth practically instantaneously, while high-resolution MRI of teeth *in vivo* in a reasonable scan time, which is limited by patient comfort and safety, is still a great challenge. For *in vivo* MRI of teeth a dedicated hardware is needed. This includes special dental RF and gradient coils, perhaps even a new magnet design. A special care must be taken also in optimization of an imagining sequence used. For the targeted tissue (dental pulp), the sequence should provide the best compromise between the resolution and scan time. As human teeth are relatively small and the resolution needs to be high, there is perhaps even a need for a stronger magnet, not only that its shape has to be adjusted for dental applications. There are no such systems on the market yet and all *in vivo* studies were done on convectional clinical scanners with or even without dedicated coils. One such study was done on a 1.5 T clinical scanner using 3D RARE with a scan time of 8 minutes, which enabled 3D visualization and quantification of caries lesions and dental pulp *in vivo.*^25^ The resolution obtained was 300×300×300 μm^3^, which is relatively low compared to what can be obtained *in vitro* in high-field high-resolution NMR magnets. Unfortunately, these magnets are expensive in comparison to permanent or resistive magnets; however, they have numerous advantages. Not only that their magnetic field is high, they also produce very stable and extremely homogeneous magnetic field. So they are practically the only efficient solution for high-resolution MRI of teeth. An example of a dental study using high-field NMR magnets was done by Baumann *et al*.^28^ who used 7 T magnet to obtain high-resolution images of a root canal system with isotropic resolution of 98 μm. Presently, more feasible *in vitro* studies are less relevant for the clinical use. However, they represent a good platform for a new method development. With ever increasing development of clinical MRI hardware some of them may soon become clinically applicable. In this study we have shown that MR microscopy of teeth enables spatial visualization of a dental pulp and root canal system with high accuracy. *In vivo* use of this method would represent a major breakthrough in dental radiology from which endodontic, periodontal and odontogenic treatments would benefit.

## Conclusions

X-ray radiography is despite its harmful effects and a lack of accuracy still a method of choice in the assessment of dental pulp morphology and a pulp canal system. As it is a projection technique, it is prone to overlapping of anatomical structures, which makes its diagnostic accuracy very limited. We have shown in our study, that these deficiencies of radiography can be overcome by the use of high-resolution MRI. MRI is harmless and enables acquisition of 3D images of soft dental tissues with a high spatial resolution. Post-processing of 3D MRI data enables precise visualization of all dental pulp anatomical features either by a sequence of 2D slices in an arbitrary orientation or by volume rendered images from arbitrary viewpoints. With ever increasing development of clinical MRI hardware, the result of this *in vitro* study may soon become clinically applicable.
